# Assessing the Feasibility of a Faith-Based Colorectal Cancer Education and Screening Intervention for Latino Men in Pennsylvania

**DOI:** 10.1177/15404153231214714

**Published:** 2023-11-14

**Authors:** Raffy R. Luquis, Sol M. Rodriguez-Colon, Sarah Ines Ramirez, Eugene J. Lengerich

**Affiliations:** 1 School of Behavioral Sciences and Education, 43324 Penn State Harrisburg, Middletown, PA, USA; 2 198599 Penn State Cancer Institute, Penn State University, Hershey, PA, USA; 3 Department of Family and Community Medicine, 12310 College of Medicine, Penn State University, Hershey, PA, USA; 4 Department of Public Health Sciences, College of Medicine, Penn State University, Hershey, PA, USA

**Keywords:** Hispanic-Americans, colorectal cancer, Latino men, cancer screening

## Abstract

**Introduction:** Limited health knowledge, literacy, engagement in preventive health services, participation in health promotion behaviors, and cultural factors place Latino men at high risk for colorectal cancer (CRC). This pilot study aimed to determine the feasibility and acceptability of a faith-based cancer education intervention focusing on Latino men between 45 and 74 years old. **Methods:** This pilot study used a single group pre- and post-intervention research design to compare changes in knowledge, perceived benefit of screening, perceived susceptibility and severity of CRC, and the completion of CRC screening after the intervention. **Results:** In this study, Latino men were willing to participate in a CRC educational intervention supported by a faith-based institution. The participants had limited knowledge about CRC, yet most recognized that screening is beneficial and that getting CRC is serious. Sixty percent of the participants completed the fecal immunochemical screening test, which showed that the intervention impacted the screening uptake among this group. **Conclusion:** The findings of this study support the further development of faith-based interventions focusing on Latino men.

## Introduction

Limited health knowledge, literacy, engagement in preventive health services, participation in health promotion behaviors, and cultural factors place Hispanic/Latino^1^ men at high risk for colorectal cancer (CRC) (American Cancer Society [ACS], 2021; Carrion et al., 2013; Oduro et al., 2012; Sobralske & Lee, 2010). In the United States (U.S.), CRC is the second most common cancer and the second leading cause of cancer-related deaths among Latino males (ACS, 2021). The CRC screening rate among Latino men 45 years old and older (45%–62%) is lower than non-Latino white men of the same age (58%–68%) (ACS, 2021). Moreover, the CRC screening rate among Latino men over the age of 45 varies by subgroup, with Mexican reporting the lowest rate (45%) followed by Dominican (48%), Cuban (50%), Central/South American (54%), and Puerto Rican (62%) (ACS, 2021). Using data from the 2010 and 2015 National Hispanic Interview Survey, Calderón-Mora et al. (2022) found similar rates of ever having a CRC screening test among Latino subgroups, with Mexican reporting the lowest rate (50%) and Puerto Rican the highest (62%). Consequently, Latinos have the highest risk for advanced-stage CRC diagnosis compared with other racial/ethnic groups (Mojica et al., 2018; Ou et al., 2019). Although the CRC mortality rate among Latino men (13.7 per 100,000) is slightly lower than the rate among non-Hispanic White (15.8), in some states (e.g., Texas, California), Latinos CRC mortality rates approach or surpass those of non-Hispanic White men (ACS, 2021). Likewise, results from previous studies have shown a difference in mortality rates among Latino subgroups compared to non-Hispanic Whites (Chien et al., 2005; Pinheiro et al., 2017; Soto-Salgado et al., 2009). Finally, the overall 5-year survival rate for Latinos diagnosed with CRC at a localized stage is 90%; however, the survival rate declined to 71% and 15% for those diagnosed at regional and distant stages, respectively (ACS, 2021). Developing, implementing, and evaluating health promotion initiatives that address CRC screening focusing on Latino men is essential.

According to Krogstad et al. (2023)**, **over two-thirds of Latinos in the U.S. reported being affiliated with a religious faith, with Catholics remaining the largest religious group among them. The percentage of Latinos who reported being affiliated with a religious faith is higher among those who were older than 50 years of age, spoke mainly Spanish, and were foreign-born. Given the importance of religion among this group, faith-based organizations provide a perfect setting for the incorporation of health promotion programs and disease-preventive interventions. Moreover, previous studies have shown that faith-based interventions promote and increase cancer screening behaviors among Latinos, especially among women (Allen et al., 2014a, 2014b, 2016a, 2016b; Hou & Cao, 2018; Schwingel & Galvez, 2017). According to Allen et al. (2014a), Latinos expressed a deep connection between their religious beliefs and health and emphasized that this connection promotes positive health behaviors and healthcare utilization. Similarly, Schwingel and Glavez (2017) found that Latinos consider the church a trustworthy, natural, and authentic setting to engage in health promotion programs. Promising faith-based interventions have included group educational sessions, behavioral goal setting, support from the priest/pastor, and recruitment of Latino church participants (Allen et al., 2014a, 2016a; Hou & Cao, 2018; Schwingel & Galvez, 2017). Results from a qualitative study on preventive behaviors among Latino men showed that their religious beliefs influenced their behavior, and religious organizations provided a vital support system within their community (Luquis, 2019). Hence, a study that assesses the feasibility and acceptability of a faith-based intervention to increase CRC screening among Latino men is warranted. Finally, Mojica et al. (2018) concluded that using culturally appropriate strategies should include educational strategies that stress the importance of cancer screening, explore and address barriers to screening, and provide Latino men with the opportunity to ask questions and support to uptake screening. However, the literature needs to be more extensive in the number of studies with enough representation of Latino men in their sample; hence, more research is needed to investigate the effectiveness of interventions that promote CRC screening among them.

### Purpose

This pilot study aimed to determine the feasibility and acceptability of a faith-based cancer education intervention focusing on Latino men between 45 and 74 years old. This pilot study aimed to answer the following: a) would Latino men participate and remain engaged in a faith-based CRC screening intervention? b) what is their knowledge, perceived benefit of screening, perceived susceptibility, and severity of CRC? and c) would the intervention lead to an uptake of CRC screening among this group?

## Methods

### Sample

Forty-five Latino men were recruited through six faith-based organizations (i.e., Catholic and Christian churches) serving the Latino community through six central and eastern Pennsylvania counties. Participant recruitment was done in partnership with church and community leaders between April 2022 and March 2023. The research team recruited the Churches with the help of the Hispanic/Latino Cancer Community Advisory Board (CAB) (Penn State Office for Cancer Equity, 2023). The selected counties are part of the Penn State Cancer Institute (PSCI) catchment area (Manne et al., 2023). Interested participants were informed of the purpose of the CRC educational intervention, the CRC screening test, the scheduled date and time for the education session, and the location (i.e., after Sunday church service, evening time). A research team member screened each participant for eligibility on the day of the educational session. Criteria for inclusion into the study were: men who self-identified as Latino, between the ages of 45 to 74, were Spanish speaking or bilingual (Spanish and English speaker), had no history of CRC, were capable of completing the pre-and post-intervention surveys, and the at-home collection fecal immunochemical test (FIT) CRC screening test (Quest Diagnostics, n.d.).

### Measurements

The study used a mixed-method research approach. The study used a single group pre- and post-intervention research design to compare changes in knowledge, perceived benefit of screening, perceived susceptibility and severity of CRC, and the completion of CRC screening after the intervention. In addition, participants were asked to complete four open-ended questions to assess the acceptability of the intervention at the end of the educational session. The first author developed the pre- and post-intervention surveys using existing instruments used in previous studies (Luquis, 2019; Luquis & Kensinger, 2019). The pre-intervention survey included ten questions to elicit demographic information and nine regarding health status and screening behaviors. In addition, participants were asked to choose one answer on eight multiple choice questions regarding knowledge of CRC, including risk factors (e.g., What foods may cause colon cancer?), screening (e.g., At what age is colorectal cancer screening recommended to begin?), symptoms (e.g., Which of these is not a symptom of colon cancer?), and incidence among Latinos (e.g., Which of the following is true about colorectal cancer in Latino men?). Participants were also asked to respond to four statements to determine their perceptions of screening benefits, CRC susceptibility and severity, and screening self-efficacy. Perception of benefits was measured by asking the participants whether they agreed with the benefits of early CRC screening using a 5-point Likert scale (1 = strongly disagree, 5 = strongly agree). Self-efficacy was measured by asking the participants how likely they would be able to complete the home CRC screen test using a 5-point Likert scale (1 = non-likely, 5 = very likely).

Similarly, perception of susceptibility was measured with a single item (i.e., compared to other men your age, what would you say your chances are for developing colorectal cancer?) on a 5-point Likert scale (1 = much lower than average, 5 = much higher than average) and perception of severity was assessed by asking participants to respond to statement, “compared to other men of your age, getting colorectal cancer would be a serious problem?” on a 5-point Likert scale (1 = strongly disagree, 5 = strongly agree). The instrument was translated from English to Spanish and back-translated by research team members to ensure all participants could complete it in their preferred language. The post-intervention survey also included questions assessing cancer knowledge, barriers to screening, perceptions of screening benefits and perceived cancer susceptibility and severity, and self-efficacy, and asked participants to self-report their completion of FIT screening and to report any issues completing the screening or reasons for not completing it. At the end of the education session, each participant was asked to answer four open-ended questions, including what they liked most and least about the educational session, whether they would recommend it to other men, and anything else they wanted to share about the experience.

### Data Collection

The University's Office of Research Protection approved the study before the recruitment of the participants. Each eligible participant read and signed the consent form and completed the pre-intervention survey before participating in the CRC educational session. They were asked to complete the post-intervention survey a month after the educational session. Ineligible participants were allowed to stay and listen to the CRC educational session.

Research team members developed the culturally informed CRC educational session guided by the health belief model (HBM) (Champion & Skinner, 2008). According to the HBM, individuals perceived seriousness, susceptibility, benefits, barriers, and cues to action can be used to explain whether persons take actions to prevent and engage in screening behaviors to improve their health. The HBM has been effectively used to develop interventions that address CRC screening among the Latino population (Hou & Cao, 2018; Juárez-Carrillo et al., 2017; Lairson et al., 2018). The intervention was delivered in Spanish by a health education specialist. It included education about the digestive system, CRC risk factors, the seriousness of CRC, the incidence and prevalence of cancer in the Latino population, and information on the benefits of cancer prevention and screening, survival rate, and treatment.

In keeping with the ACS (2020) recommendation for CRC screening starting at 45 years old, each participant received a FIT at the end of the education session. Participants received written instructions in English and Spanish on collecting the sample and completing the FIT**.** They were instructed to mail the completed FIT to the laboratory within a week, free of charge. Each participant received a $25 gift card at the end of the education session. A week following the education session, participants received a phone call reminding them to complete the FIT and answer any questions they may have had about it. To maintain confidentiality, each participant was assigned a unique study identification number to match the pre- and post-intervention surveys, session evaluation form, and FIT lab results. Using the identification number, a research team member received the lab results and sent them to each participant via mailed letter. Information on each participant's healthcare provider was not collected; hence, the research team could not contact each healthcare provider with the results. However, participants were encouraged to share their FIT results with their primary healthcare provider and consult them regarding follow-up testing, as necessary. For those participants without a healthcare provider, the research team was ready to provide referrals to local healthcare providers if needed in the case of positive CRC screening results. Post-intervention surveys were mailed to participants a month after completing the education session. The letter instructed the participants to return their completed surveys in the included business reply (i.e., no postage necessary) envelope.

### Data Analysis

Data were entered into the Research Electronic Data Capture (REDCap) system. Descriptive statistics, including frequency distribution, mean, and standard deviation, were used to examine participants’ demographic characteristics, health status and screening behaviors, knowledge questions, and CRC perceptions. Participant knowledge was analyzed by calculating a composite score by summing the number of correct answers. The screening completion rate was calculated based on the number of completed screening FITs reported by the lab compared to the total number of participants. All analyses were performed using the statistical software SPSS version 28. The responses to the open-ended responses were summarized.

## Results

### Sample

Of the 54 men who attended the CRC education session across six locations, 45 were eligible and participated in the study. The number of participants at each education session ranged from three to twelve men. In three Christian churches, the pastors helped recruit the participants, encouraged them to attend, and participated in the education session. In the first Catholic Church, a community member helped with the recruitment. In contrast, in the second Catholic Church, participants were recruited through an established group in which Latino men and women actively participated (i.e., Movimientos de Retiros Parroquiales Juan XXIII). Participants were recruited in the last Catholic Church by placing an announcement in the church bulletin with the priest's permission.

Participants were 45–77 years old (median = 58 years old), married/living with a partner (84%), employed full-time or self-employed (68%), and earned less than $35,000 per year (74%). Almost one-third of them (36%) completed some high school education or less education, and 31% had a high school education. Most participants were born outside of the U.S. (91%), with 41% identifying as being from Puerto Rico and 41% from the Dominican Republic. Almost all indicated they primarily spoke Spanish at home (89%). Almost two-thirds indicated they did not speak English well/not at all (67%) (See Table 1).

**Table 1.** Participant Sociodemographic Characteristics (N = 45).

	n	%
Age (in years)		
45–55	22	50
55–65	11	25
65 and above	11	25
Mean (SD) = 57.7 (8.8)		
Education Attainment		
Some high school or less	15	36
High school/GED	13	31
Some college/Associate degree	9	21
Bachelor's degree	5	12
Marital Status		
Married/living with a partner	38	86
Divorced/legally separated	4	9
Never Married/widowed	2	5
Employment		
Full-time	20	47
Part-time	2	5
Self-employed	9	21
Not Employed	12	28
Income		
Less than $15,000	6	16
$15,000 to less than $20,000	9	24
$20,000 to less than $25,000	4	10
$25,000 to less than $35,000	9	24
$35,000 to less than $50,000	7	18
$50,000 or more	3	5
Hispanic Origin		
Puerto Rico	18	41
Dominican Republic	18	41
Central & South America	4	9
Mexico	3	7
Cuba	1	2
US Born		
Yes	4	9
No	38	91
**Primary l**anguage spoken at home		
Spanish	39	89
Both Spanish and English	5	11
**How well they s**poke English		
Very well	1	2
Well	14	31
Not well	22	49
Not at all	8	18

*Note*. percentage based on those participants that answered the questions, total percentage may not add up to 100 percent due to rounding, missing data was excluded.

### Health Status, CRC Screening, Perceptions, and Knowledge

Most participants self**-**reported their health status as “good/fair” (64%). Most of them had health insurance (76%), almost two-thirds had a healthcare provider (i.e., private or community health clinic) (63%), and received a routine health check-up within the past year (65%). Most participants (91%) reported not being screened for CRC within the past year; however, 40% self-reported that they had had a sigmoidoscopy or colonoscopy in the past, with the majority of them within the past two or more years (see Table 2).

**Table 2.** Health Care and Preventive Behaviors.

	n	%
Health Status		
Excellent	7	16
Very good	9	20
Good	18	40
Fair/poor	11	24
Health Insurance Coverage		
Yes	34	76
No	11	24
Place for Health Care Provider		
Private Practice	15	35
Community Health Center or Clinic	12	28
ER	5	12
None	10	23
Last Time Received Routine Check-up		
Within past year	28	65
Within the past two years	9	21
Three years or more	6	14
CRC Screening Past Year		
Yes	4	9
No	38	91
Colonoscopy or Sigmoidoscopy		
Yes	18	40
No	27	60
Time of Colorectal Cancer Exam		
Within the past year	3	18
Within the past 2–3 years	5	29
Within the past 5 years	3	18
Past 10 years or more	6	35

*Note*. percentage based on those participants that answered the questions, total percentage may not add up to 100 percent due to rounding, missing data was excluded.

Most participants strongly agreed or agreed that CRC screening is beneficial (96%), that getting CRC is a very serious problem (95%), and that they were very likely or likely to complete the home CRC screening test (90%). However, 46% believed that they have an average, and 28% believed they have a lower or much lower than average risk of developing CRC (see Table 3). Most participants had limited knowledge about CRC. Over 60% of the participants correctly answered questions related to food that may cause cancer, lifestyle choices to decrease CRC risk, and the difference between the incidence of CRC between men and women. Approximately half knew the risk factors for developing CRC. However, less than 40% knew the ages to begin and stop CRC screening, possible symptoms, and incidence of CRC among men. The mean knowledge score was 4.16 based on those who answered all the questions (n = 43) (see Table 4). Only eleven participants returned the post-intervention survey in the mail after several phone calls and reminders; as such, data from the post-intervention survey were not included in this analysis.

**Table 3.** CRC Perceptions of Screening Benefits, Risk, Seriousness, and Likelihood of Action.

Statement	n	%
CRC screening is beneficial		
Strongly agree	35	88
Agree	3	8
Neutral	1	2
Disagree	1	2
Risk for developing CRC		
Much higher	7	18
Higher	3	8
Average	18	46
Lower	9	23
Much lower	2	5
CRC seriousness		
Strongly agree	28	70
Agree	10	25
Neutral	2	5
Likelihood of completing FIT		
Very likely	17	45
Likely	17	45
Neutral	3	8
Very unlikely	1	2

*Note.* percentage based on those participants that answered the questions, total percentage may not add up to 100 percent due to rounding, missing data was excluded.

**Table 4.** Correct Responses to the Knowledge Questions About CRC Risk, Screening, and Signs.

	Pre-survey	
Question	n	%
Risk factors for developing colon cancer	23	54
Age to begin CRC screening	16	37
Age to stop getting CRC screening	14	33
Food that may cause cancer	35	81
Lifestyle choices that decrease the risk for CRC	31	72
Symptoms of CRC	14	33
Incidence of CRC among men	12	28
Incidence of CRC men vs. women	28	65
Knowledge composite score	1–8	
Mean (SD)*	4.08 (1.49)	
Range	1–7	

*Note*. percentage based on those participants that answered the questions, total percentage may not add up to 100 percent due to rounding, missing data was excluded.

Finally, while twenty-eight participants reported completing and returning the FIT to the lab, we received the results from 27 (60%). Twenty-six of the test results were negative, and one was inconclusive. Further analysis showed that two-thirds (65%) of those who completed the FIT CRC screening test were between 45 and 60 years old. Moreover, 24 of those who completed the FIT (89%) self-reported in the pre-intervention survey that they had not had a CRC screening in the past year (not in the table).

At the end of the intervention, participants responded to four open-ended questions regarding the educational session. Forty-three of the participants responded to the questions. The majority of them were very satisfied with the information presented regarding CRC. For example, one participant stated “Me gusto todo, porque aprendí lo que no sabía” (I liked everything because I learned what I did not know), other participants stated, “Toda la informaciones estaban muy importantes” (all the information was very important), and “nos informó cómo cuidarnos y de lo importante de comer una dieta sana” (he informed us how to take care of ourselves and the importance of eating a healthy diet). Several of them appreciated receiving the information in Spanish. Almost all of them said they would recommend the intervention to other men. When asked if they did not like something, one man stated that he did not like the length of the pre-intervention survey. Finally, several men indicated they liked the presentation because it was interactive and allowed them to ask questions. Some of them stated they were thankful for the opportunity to participate, and some participants wanted information on other topics, such as prostate cancer.

## Discussion

Findings from a recent systematic review of faith-based health interventions for Latinos showed that partnerships between public health entities and faith-based organizations could be an effective way to reach underserved Latinos (Allen et al., 2014a, 2014b, 2016a, 2016b; Aumiller et al., 2013; Derose & Rodriguez, 2020; Hou & Cao, 2018; Kretzler et al., 2022; Schwingel & Galvez, 2017). This pilot study showed that recruiting Latino men to participate in a faith-based CRC educational intervention is feasible when the intervention is supported by the pastor/priest and a church group and occurs immediately after a church service. Based on a systematic review of the literature, Derose and Rodriguez (2020) suggested that more extensive randomized controlled trials are needed to evaluate the effectiveness of these interventions among Latinos and those implemented in the African American community.

Secondly, in keeping with previous findings, most of the participants in our study had limited CRC knowledge (Colón-López et al., 2023; Espinoza-Gutarra et al., 2023; Gonzales et al., 2023; Ou et al., 2019). While participants in our study were aware of CRC risk factors, including nutrition and lifestyle choices, they needed to know more about the recommended screening age and symptoms associated with CRC. Since the age recommendation to begin screening was changed from 50 to 45 within the past 5 years (ACS, 2020), participants were unaware of that change. However, the low post-intervention survey completion precludes us from determining whether the intervention impacted this group's CRC knowledge. While participants who completed the post-intervention survey received a $10 gift card in the mail after completion, more was needed to motivate them to complete it. In addition, completion of the post-intervention survey via phone or in person was not feasible during this study.

While most of our participants recognized that CRC screening is beneficial and the seriousness of getting CRC, most perceived their risk of getting CRC as average or less than average; similarly, while most indicated they were likely to complete the FIT, and each participant received several reminder calls encouraging them to complete it, only 60% did. This result showed that the intervention impacted the uptake of CRC screening among this group. While the majority of those who completed the FIT self-reported that they had not had a CRC in the past year, since the research team did not have access to the participant's medical records, it is impossible to corroborate the self-report CRC screening information to determine the true impact of the intervention. The CRC screening result is similar to the CRC screening rate previously reported (ACS, 2021; Castañeda et al., 2019; Mojica et al., 2018; Viramontes et al., 2019), however, it is possible that their pre-intervention perceived susceptibility, average or less than average chance of developing CRC, may have contributed to the lower FIT completion rate. In addition, since 40% of them reported having a CRC screening within the past few years, they possibly did not see the need to complete the FIT. Colón-López and colleagues (2023) found that while Latinos acknowledged being at risk for CRC, they would not go to their primary healthcare providers if asymptomatic and delayed care when symptomatic due to fear about a diagnosis. Ou et al. (2019) also found that CRC screening adherence was higher among Latino women than men.

Moreover, previous studies showed that whether Latino men take preventive action depends on their views on the disease, prevention, fear of the results, and cultural values (Luquis, 2019). In the present study, most of those who completed the FIT screening test were under 60, which indicates that the intervention motivated these men to engage in prevention behaviors since they perceived the benefit of screening and the seriousness of CRC. Since the present study utilized follow-up phone calls to encourage FIT completion among participants, future interventions should examine other strategies to encourage participation in CRC screening among this group. For example, Colón-López et al. (2023) recommended that including men who have gotten screened, found cancer, and were successfully treated may increase participation and screening among Latino men. Future studies should consider including the participants’ healthcare provider or a clinical partner to ensure that the men complete the CRC screening, as they may be more willing to follow the recommendation from a healthcare provider (Luquis, 2021; Mojica et al., 2023). In addition, sharing the FIT results with the participants’ healthcare providers would ensure that they received the follow-up screening test (i.e., colonoscopy) if needed (Mojica et al., 2023).

Several limitations must be considered when examining the results of this study. First, although we received the help of CAB members in the recruitment process, the participation of Latino men was lower than expected, which limits the generalization of the results. Future studies should try other strategies to encourage Latino men's participation in this intervention. Since the recruitment efforts were concentrated in six specific counties, the results cannot be generalized to other Latino men throughout the state. The self-selection nature of the study further limits the generalization of the results, as participants may have had a self-interest and were influenced by their church leader into participating in the intervention.

Moreover, fewer participants completed the post-intervention survey, which prevented us from generalizing regarding the impact of the intervention on the participants’ knowledge and perceptions regarding CRC. Finally, while one of the FIT results was inconclusive, we were still looking for positive results. The small sample size may preclude us from identifying positive FIT results among this group.

## Conclusion

The findings of this study further support the development of faith-based interventions focusing on Latino men, as it showed that men were willing to participate and were very satisfied with the educational intervention. The results also showed that Latino men acknowledge the benefit of CRC screening and that this intervention might increase CRC screening via FIT among this group. The results of this study could support further development of a comprehensive Latino men's community intervention for promoting CRC screening and cancer prevention. The development and implementation of CRC education and screening intervention should include information about other cancers, chronic diseases, and health issues the men in our group expressed interest in (e.g., prostate cancer). Since risk factors for CRC include modifiable factors (i.e., diets, weight, smoking), medical history (i.e., type 2 diabetes, chronic inflammatory bowel disease), and hereditary factors (i.e., personal, family history), additional information about these may allow participants to have a broader understanding of CRC and engage in preventive actions. In addition, future interventions should include church leaders in recruitment efforts and educational sessions, which should occur immediately following church service. Similarly, future interventions should include testimonials from Latino men screened for CRC who have been successfully treated for CRC, as they can serve as role models for this group. Finally, interventions should consider conducting post-intervention surveys by phone as it will provide a personal touch with the participants, an essential aspect of the Latino culture, which may help increase the post-intervention survey completion rate.

## Notes

1. Latino(s) is used throughout the paper to refer to persons who self-identified as Hispanics or Latinos.
